# Commentary on Highly Successful Female Educational Psychologists: Equity and Intersectionality in Success Definitions

**DOI:** 10.1007/s10648-023-09727-3

**Published:** 2023-01-26

**Authors:** Natalia Kucirkova

**Affiliations:** 1 Norwegian Centre for Learning Environment and Behavioural Research, University of Stavanger, NO-4036 Stavanger, Norway; 2 The Open University, Milton Keynes, UK

**Keywords:** Intersectionality, Equity, Gender, Women in science, Scholarly success

## Abstract

Despite the international focus on validation and success indicators of academic work across disciplines, examples of accomplished educational psychologists and their personal stories have been limited in the literature. Prinz et al. (2021) interviewed Five Highly Successful Female Educational Psychologists and offered a content analysis of their success stories, including the academics’ strategies and trademark characteristics. In this Commentary, I expand on their findings in light of equity and intersectionality perspectives on academic success. I problematise academic success indicators (publication records and impact) with reference to literature on gender disparities in publication metrics and lack of inclusivity in generic measures of academic success. I outline how individual success narratives intersect with our collective responsibility for higher wellbeing and professional recognition of all colleagues. I argue that the equity and intersectionality perspectives are fundamental to inclusive narratives of success and highlight the power structures that have historically impeded access of diverse and minority scholars to top academic positions. I conclude with four recommendations for addressing the persistent structures of inequities in academic career opportunities.

## Introduction

The realities of modern academia have been under spotlight in the past five years, with an increased public, as well as scientific, interest in the ways in which academic success is defined and achieved (Sousa & Clark, 2018; Gewin, 2022). The portrayals of successful professors (e.g., Prinz et al., 2021) as well as top early career researchers in educational psychology (Kiewra, Luo & Flanigan, 2021) in this journal tap into this interest. The studies follow a typical psychological perspective by focusing on the success of individual researchers. I supplement their insights with a perspective from feminist and sociological theories for a theoretically oriented discussion on supporting careers and improving minority representation in educational psychology and academia more broadly. I frame my Commentary in the intersectionality perspective (Cho, Crenshaw & McCall, 2013) that highlights how power and intersecting identities are embedded in social relations. My aim is to encourage a collective reflection on the ways in which stories of individual achievements can serve as a backdrop to inclusive and equitable narratives of what it means to succeed in today’s academia.

### Individual Success Stories

Prinz et al. (2021) interviewed five leading female educational psychologists and Kiewra et al. (2021) six early career award-winning educational psychology scholars (three male and three female) in order to identify the work practices that have helped with their success and high productivity rates. I highly admire and respect all the interviewees selected for the two studies, and agree with the authors that their success stories speak loudly to all educational psychologists.

Although ‘alternative attempts to identify the most productive educational psychologists are simply additional mirrors provided to the already excessively vain’ (Hsieh et al., 2004, p. 340), and documentation of productivity might ‘run the risk of over-self-promoting’ (ibid, p. 341), success narratives are important for universities’ external engagement with the public as well as internal processes of identifying suitable mentors. Productivity studies are today supplemented with automated services (e.g. https://research.com/) that rank ‘best scientists’ in each discipline and provide immediate productivity measures by displaying the number of citations, downloads and Altmetric scores. Some might consider these as digital brag sheets and some as new mirrors of individual academics’ success. What is certain is that the lived experiences of productive researchers are missing, and Prinz and Kiewra et al. (2021) address this gap with insights into the factors that affect the productivity of most successful educational psychologists.

Exchange of effective strategies, tools and techniques might advance individual effectiveness, and support success narratives with mapping and increasing individual skills and expertise. As such, Prinz and Kiewra et al. (2021) add to the growing efforts intended to upskill female academics in effective leadership styles and professional training opportunities targeting academic women (e.g. Women’s Leadership Projects at individual institutions, such as the Michigan State University in the USA, the national Aurora programme in the UK or initiatives connected to Athena SWAN charter, a framework dedicated to transforming gender equality within higher education internationally).

Similarly to Kiewra et al. (2021), I recognise the case of increasing advantages for successful scholars and consider both their and Prinz and Kiewra et al. (2021) portrayals of successful female psychologists as useful case studies for the ongoing discussions of diversity in academia. My aim is to expand the academic diversity discussions as I delve deeper into systemic disparities that generate forms of social capital that become unevenly distributed across academic groups.

The ‘She Figures’ 2012 report of the European Commission and the ‘Staying Power Report’ (2019) reveal that there are dramatically few Black female professors across European and UK universities. The absence of female professors representing first-generation status, disability, working class, motherhood, English spoken as a third language and education from non-Anglo-American countries further reveals the gaps at the top academic positions. It is important that we, as a field, collectively engage with this reality. In what follows, I cut to the core of how inequitable professional narratives at the university workplace are embedded in implicit and explicit narratives of academic success.

### What Is Academic Success?

To discuss the most *successful* female academics, we need to define what success means for women working at higher education institutions and universities. Prinz et al. (2021) defined success as ‘general scholarly success (publication quality, influence on practice, etc. besides publication quantity’ (p. 767). This focus is a welcome and long overdue antidote to publication counts, which, although used by some hiring committees and science evaluators, have been repeatedly criticised by interdisciplinary and international scholars for being a poor certification of scientific merit (e.g. Trenchard, 1992; Feller, 2004; Zuo & Zhao, 2021). Prinz et al. (2021) established the most successful female educational psychologists through an open call for nominations via social media of two key professional networks. Considering the importance of peer esteem and reputation in academic circles, this is a suitable democratic method for establishing who is perceived as successful in current educational psychology circles.

In interpreting Prinz et al.’s (2021) interview findings, I draw on the knowledge accumulated during the two-year-long project ‘Inspirational Women in Academia’ (Kucirkova & Fahad, 2022). As part of the project, Loleta Fahad and I interviewed thirteen women working at research-active universities either in a research or administrative capacity at some of the world’s most prestigious Higher Education institutions: the Harvard Graduate School of Education, Oxford University, University College London, McGill University and Berkeley School of Education. These women were identified through a snowball sampling strategy whereby we started with one interviewee we considered to be our academic female role model, who then recommended another colleague who she considered to be an inspirational academic and that colleague was interviewed and asked for a follow-up recommendation and so on. A sample of inspirational academic women can’t ever be complete or objective, but our informal peer nomination method enabled us to gain some deep and authentic insights into the factors that some academic women associate with their successes. The interviews were conducted by Zoom and automatically transcribed by the Zoom software. We analysed the transcripts for recurring patterns and discussed them in the book in relation to different identities performed by women in the academic workplace. The results have been summarised in a book titled ‘Inspirational Women in Academia Supporting Careers and Improving Minority Representation', and in this Commentary, I highlight the project’s findings that supplement Prinz et al.’s (2021) insights. In accordance with our ethical clearance protocol from the Norwegian Centre for Research Data (NSD), we followed the participants’ own preferences for either being named or remain anonymous. The selected quotes are anonymised and used for illustrative purposes.

One of Prinz et al.’s objectives was to ‘understand the circumstances and strategies that buffer successful female educational psychologists against career setbacks’. I am interested in the ways in which individual success stories could act as bridges and frontiers in mediating the path for all women and address the barriers to greater representation of minority women at higher ranks, and especially at top-performing institutions. My aim is to expand gender-neutral descriptions in performance narratives, challenge the barriers to professional participation of minority-status women and open up productivity studies to new understandings of academic success.

#### Traditional Criteria of Success

Although Prinz et al.’s (2021) solicitation of nominations only mentioned ‘contributions, visibility and influence’, the top nominees all had very high research outputs and large influence on practice. Prinz et al. (2021) discuss the academics’ productivity and the strategies the academics used for achieving it. I supplement their discussion with a reflection on how we collectively approach three factors aiding and reflecting productivity: publication metrics, influence on practice and research specialisation. The three factors were mentioned but not critically engaged with by the authors and I discuss how these aspects feed into collective definitions of academic success, with an eye towards gender and intersectional disparities.

#### Gender Disparities in Academic Productivity

Gendered differences in productivity metrics, research specialisation or possibilities for large-scale influence on practice reflect the embedded inequalities in academic productivity, including the high correlations between academic women working more part time (Misra, Lundquist & Templer, 2012), in more teaching positions (O’Meara, Kuvaeva, Nyunt, Waugaman & Jackson, 2017) and service work (Guarino & Borden, 2017), as well as academic women experiencing less uninterrupted research time (Alexander & Shaver, 2020; Pebdani, Zeidan, Low & Baillie, 2022) and greater work/life conflicts because of being the primary caregiver for children (Tower, 2015). Gender remains a barrier for the transition from postgraduate to higher levels of academic career, as exemplified by data from Australian science students, who attributed the transition barriers to the incompatibility between motherhood and a successful career in academia (Crabb & Ekberg, 2014).

The causes for gender disparities in science were attributed to women’s choices, which are subject to constraints and sociocultural influences (Ceci, Williams & Barnett, 2009). I zoom in on the constraints and sociocultural influences in relation to three factors that influence productivity: publication metrics, research specialisation and influence on practice.

#### Publication Metrics

Prinz et al. (2021) recognise gender *differences* in academic careers, including gender-role expectations, gender-biased discrimination, and gender equality engagement. Gender *disparities* in favour of men in publication rates have been documented in individual countries at a given point in time (e.g. in Mexico, Canada and USA by Padilla et al., 2011; Norway by Rørstad & Aksnes, 2015) as well as through historical comparisons covering 83 countries and 13 disciplines (Huang, Gates, Sinatra & Barabási, 2020). Unlike in Prinz et al.’s (2021) study, previous research compared top-performing male scholars to female scholars (Greenbaum et al. 2018). Greenbaum et al. (2018) analysed authorship in five educational psychology journals and found that top female authors are in more junior positions than top male authors and that although the representation of females in professional member organisations (AERA and APA) had increased in the span of 2004–2016, this was not reflected in female representations in editorial roles and editorial boards. Latest analyses of the impact of the COVID-19 pandemic on scholarly production further confirm the disadvantage for women, with disproportionally higher number of papers published by male authors during the first wave of the COVID pandemic (Squazzoni, 2021).

The patriarchal history of the publishing world carries not only consequences for the publication quantity to be positively skewed towards men but also measures of publication quality. Publication quality is typically measured with citation counts but these map neatly on the explanatory factors for academic career progression, such as time spent on research and international collaboration, which, even in most gender-progressive countries like Norway, are more available to men than women (Bentley, 2009). In addition, citation count is a difficult parameter of scholarly achievement because of mutual citations, self-citations and the likelihood of articles by larger teams to be more cited than single-authored articles (Anderson & Richards-Shubik, 2021). There are also more subtle disadvantages that are not directly reflected in citation numbers. For example, most journals have no effective structures for accommodating name changes in Altmetrics. Women who published papers and change their names at marriage or divorce thus face the dilemma of declaring personal change of circumstances in professional context and the burden of managing their publication record with two identities (Peterson, 2019).

#### Research Specialisation

Research specialisation is associated with higher productivity (higher number of articles) and visibility (where the articles are published and how much they are cited). Leahey, Crockett and Hunter (2008) introduced research specialisation as a way of discussing and measuring professional capital. Their longitudinal analysis showed that specialisation is positively related to productivity in female academics, but the growth of productivity was in favour of male academics. Leahey et al. (2008) note that the gender-related productivity differences don’t appear until six to seven years, leading to large gender-related productivity differences by mid-career. O’Brien and Hapgood (2012) further add that for women in science and engineering, the productivity phase coincides with the final decade of childbearing, thus excluding many women. Furthermore, career interruptions and teaching commitments, which are strongly correlated with productivity metrics, are disproportionally higher for women (see e.g. Robinson, 2011).

#### Influence on Practice

Both Prinz et al. and Kiewra et al. (2021) found that academics’ professional networks and research projects matter in influencing practice. The findings from our project and analyses from grant proposals’ review scores (Murray et al., 2016) show that an academic’s possibility to influence practice depends on institutional resource allocation that is skewed towards larger, research-oriented universities. At large and well-established research institutions, individual academics have access to more resources for writing time and can prioritise research over other obligations, which provides greater opportunities for applying for larger funding grants, which in turn tend to generate larger impact (Murray et al., 2016). Furthermore, grant award procedures have historically favoured men, with women winning fewer and lower value grants (Bornmann, Mutz, & Daniel, 2007).

Another important factor to consider is the mismatch between institutional expectations of academic impact and the incentives they use for academics to invest time in influencing practice. National research bodies vary in their conceptualisations of academic influence and impact. In the UK and Australia for example, there has been a recent emphasis on scholarly and public impact, defined as evidence-informed change in practice by the UK Research and Innovation Council and Australian Research Council. Such a change can be achieved without necessarily reporting many publications, large-scale collaborative activities or commercialisation opportunities. European institutions use diverse metrics for documenting impact (Neresini & Bucchi, 2011) and promote it both as a moral imperative as well as response to funders’ mandates. Leading European impact frameworks position participatory action research as the most desirable form of public engagement and acknowledge it is also the most time-consuming one (Farnell et al., 2020). This national and institutional variety in supporting impact work indicates the different conditions under which academics achieve success, and highlight how the road to success cannot be simplified into simple ‘will and skill’ formulas adapted from corporate leadership models (Sousa & Clark, 2018).

Indeed, the gender disparities in publication metrics impact on field and research specialisation paint a complicated picture for a collective definition of academic success. Without a doubt, publications in reputable journals and citation counts are important part of evaluating scientific merit. At the same time, they beg some critical questions: are they the right metrics to gauge the magnitude of academic success? If so, how do we engage with the gender disparities embedded in the metrics? Does, for example, the focus on research specialisation mean that successful academics are only the academics who influence practice through research (rather than for example, teaching, administration, public engagement, small-scale qualitative studies or advocacy)?

Prinz and Kiewra and associates did not examine how these factors co-exist with power mechanisms and how they co-act within personal, social, cultural, economic and political systems. In what follows, I examine these relationships by drawing on the intersectionality framework.

#### Intersectional Disparities in Academic Productivity

Intersectionality is an interdisciplinary critical framework that refers to the connections and dependencies between various identity types. The term was coined by Kimberlé Crenshaw in 1989 and the concept is rooted in Black feminism and Critical Race Theory. Intersectionality has been used as both a method and epistemology for interrogating multiple and complex identities and the power structures that identities are part of (see Tefera, Powers & Fischman, 2018). Intersectionality provides a vocabulary and a set of tools to engage with questions of accumulated advantages for dominant social groups, as it highlights the power dynamics and mutual influence of social positionings (Phoenix and Pattynama, 2006). Intersectionality was used in previous studies concerned with the experiences of academic women from minority groups, for example women of non-UK origin working at UK higher education (Johansson & Śliwa, 2014) or African American women in STEM (Charleston, Adserias, Lang & Jackson, 2014). In these studies, the intersectionality framework highlighted how the relationship between gender and ethnicity (rather than gender and ethnicity as separate categories) affected the experiences of female academics. An intersection lens highlights the role of power and its continuity in promoting forms of social capital that are unevenly distributed across dominant and marginalised groups. An example of social capital in academic careers is the possibility for research specialisation or the forming experience of placements, positions or research stays at top-ranking universities.

These factors were highlighted by Prinz et al. (2021) and Kiewra, Luo and Flanigan (2021) in the success trajectories of their interviewees. Kiewra et al. (2021) reflected on the extent to which they are part of ‘the cascade of increasing advantages’ and ‘cumulative benefits’ of highly success academics. I argue that these factors exemplify cumulative benefits that intersect with inequalities.

Feminist scholars have delineated the pathways that are integral to equity-oriented perspective on addressing the factors that lie at the heart of an equity agenda in academic career progression (see Collins, 2002; Beerkens, & van der Hoek, 2022), and they shaped my thinking on alternative success measures and ways of promoting them. In the final section, I draw on the feminist literature to supplement Prinz and Kiewra et al.’s (2021) recommendations for academic success with a focus on: (1) personal dispositions, (2) diverse mentorship models, (3) workload and wellbeing strategies, and (4) failure narratives.

### Recommended foci for new academic success definitions

#### Personal Dispositions of Female Leaders

Prinz et al. (2021) and Kiewra, Luo and Flanigan (2021) found some common denominators of success and trademark characteristics across their interviews with senior female and top early career researchers. Trademark characteristics, as discussed by Prinz et al., are about the unique nature of scholars’ work or how they go about their work. In our book focused on inspirational academic women, we discuss the academics’ personal *dispositions* (Kucirkova & Fahad, 2022). Personal dispositions are an individual’s perceptions of their own strengths and weakness and are related to past and future academic achievement (Larose & Roy, 1995). A good example of personal dispositions is confidence and self-efficacy beliefs, which are known to be lower in women than in men and higher in people occupying leadership positions (Howe-Walsh and Turnbull, 2016). Although these personal dispositions might be necessary in the training of all leading positions, the support for their development has been minimal in traditional academic training (Sousa & Clark, 2018), and therefore should be prioritised in future professional development of female academics. To illustrate, the notion of female self-empowerment runs through the leadership programme developed by Knipfer, Shaughnessy, Hentschel and Schmid’s (2017) that helped German female researchers to become professors while raising their confidence, self-presentation and negotiating skills.

#### Diverse Mentors for Navigating the Academic ‘Hidden Curriculum’

The term ‘hidden curriculum’ relates to the implicit and unofficial structures and routines that feed into academic skills’ development, organisation and performance of work, but remain unexplained for those who are new or unfamiliar with the academic environment. The need for support in navigating and demystifying the unwritten rules of academia is especially important for academics who have historically been excluded from academic circles. For instance, academics who were not born speaking English, the lingua franca of modern academia, or first-generation scholars, or scholars from working class backgrounds and minority groups (African, Asian, Hispanic, and Native American scholars), might need to use more cognitive resources and/or experience more stress in understanding the hidden curriculum of academia (see for example Wyche & Graves, 1992; Patton, 2004; Papaioannou, Machaira & Theano, 2013; Horn, 2017; Carter, & Craig, 2022). One of our interviewees, native English speaker but first-generation scholar from a working-class background, described how the experience of trying to understand unofficial routines and procedures has felt to her as experiencing everyday ‘tensions’ at work: ‘Tensions … because there are a lot of things that you just, you just don't understand. There are sort of unwritten rules and expectations that somehow you don't know because you haven't come from a family that has those, if that makes sense. And it's the classic situation of you don't know what you don't know’ (Kucirkova & Fahat, 2022).

The racial, gender and class gaps that start in early education (Kozlowski, 2021) speak volumes to the scarcity of diverse narratives of how what some take for granted could be navigated at higher education level, especially by voices that thus far are almost non-existent at top academic positions: Asian American, Indigenous, multiracial, and trans* men (Cabrera et al., 2022). In addition to the structural and social factors, the political factors (in terms of the cultural, religious and social heritage of academic context) influence the social pressures experienced by minority scholars. The magnitude of challenges is much larger for women from countries with less egalitarian attitudes (Inglehart, Norris & Ronald, 2003) and patriarchal societies that have historically silenced women (Dehkharghani, Maharati, Menzies & Bridges, 2022).

Another important aspect to consider is whether the focus on individual success narratives (through interviews and sharing of career stories of top performing scholars) might de-emphasise the trajectories that arise from interdependent factors and desire to achieve progress in communities that can be achieved collectively. The intersectionality lens highlights how locating success at individual level relates to power structures that carry positive consequences for some but negative consequences for other individuals. Here, I highlight the notion of accumulated benefits for those leading academics who benefitted from inspirational mentors who belong to privileged groups. There are two aspects to unpack here: the notion of one inspirational mentor being responsible for changing the career trajectory of many, and the notion of mentors from marginalised groups being responsible for the mentorship of academics from the same marginalised group. The two aspects intersect and their intersection negatively impacts minority scholars in higher positions. Namely, being the only mentor from a disadvantaged group means being frequently asked to perform disproportionally more mentoring than scholars from privileged backgrounds. By way of a quick example, consider this pertinent quote: ‘… it’s frequently on us to do even more work, to be representative, to somehow speak for all Black people’. (Dr Raychelle Burks, cited in Hannibal, 2020, when interviewed about the challenges she faces in workplace when discussing the Picture a Scientist documentary^1^.) One of our interviewees told us that while she felt passionate about the opportunity to pay back and mentor scholars whose backgrounds reflected her own marginalised origins, she saw a clear difference in the number of requests for performing this duty as a matter of course and the expectations for women at the same rank from dominant groups. This is striking not only because of the societal burden it places on already disadvantaged groups but also in terms of the disconnect between the practice and the literature that documents the benefits of diverse and multiple mentors for women in leadership positions (Rhode, 2003).

Studies show that female academics tend to select male mentors and mentors of different ethnicity (Amabisca 2005; Almond, Parson & Resor, 2021). One of our interviewees described how the possibility to consult her career choices with male mentors from different disciplines taught her efficient time-saving techniques and boosted her confidence both as an academic and as a mother. In the Royal Society’s Brief ‘Mothers in Science: 64 Ways to Have it All (Royal Society, 2011)’, the diversity of mentors and multiple pathways to tenured positions is reflected in the 64 examples of mothers-scientists. It is vital that the mentorship models promoted by senior scholars are informed by diverse experiences and that these are embedded in evidence-based institutional mentorship policies.

#### Workload and Wellbeing Strategies

In Prinz et al. (2021), Professor Eccles reveals: ‘I sometimes joke that I’ve got a socially acceptable addiction: I just love to work. There’s always some burning issue that I’ve got to deal with’ (p.778). This quote reveals two issues. First, Professor Eccles seems to justify the extreme workload with her enjoyment of the job, raising the question of whether job satisfaction should be tied in after-work hours. Second, Professor Eccles shows a clear self-consciousness regarding the amount of work required for a top academic position. Prinz et al. (2021) compare the extreme work hours noted by Professor Eccles and their other interviewees with accounts from successful male academics, who reported working ‘50-plus hours a week and do so by beginning the workday early, continuing to work in the evening, and working on the weekend’ (Prinz et al., 2021, p. 785). The authors do not highlight extreme workload as an undesirable phenomenon but discuss it under time management strategies designed to cope with the workload, such as reducing teaching and administrative responsibilities, sharing family and household chores with partners or professional staff. In interviewing the top-performing male psychologists, Kiewra and Creswell (2000) note that the male academics reported having satisfying home lives and spoke of great enjoyment of their work. Kiewra and Creswell reflect on this finding by suggesting ‘Perhaps the stability of a university position makes extreme sacrifices less necessary’, and ‘at a larger level, we wonder how important is having a supportive spouse and family’. I wonder about this too, particularly in relation to the equity agenda. If we agree that an academic career should be considered a job and not a mission, then we should not promote time management strategies for a 50-plus hours working week but rather challenge them as a pursuit that risks compromising the academics’ own wellbeing. There is a danger to normalise a working culture where extreme workload is associated with success and where those who succeed are those who prioritise research time above anything else.

One way of normalising high workload is the promotion of benefits it provides, including the enjoyment of ‘following your passions’. Unintentionally, this career advice, which was alluded to by the successful psychologists, might undermine one’s chances for developing broader and sustainable interests (O’Keefe, Dweck & Walton, 2018) and compromise rather than advance work-life balance. Our interviewees had hobbies and interests outside of work and it was these ‘side-hustles’, and a passion for their pursuit, that allowed them to better balance up the professional/personal lives. The need for changing the academic working culture was advocated by all the inspirational academic women we interviewed, who were concerned about the greater incidence of mental health issues in pursuing top academic positions. Greater prevention measures, especially in terms of promoting a better work/life balance, mental health and wellbeing and reducing publication pressures, were clearly indicated as necessary for more equitable academic spaces.

Modelling of extreme workload by senior colleagues is particularly problematic for early career researchers or non-tenured faculty staff, many of whom perceive time management strategies as a survival strategy in a highly competitive workplace: ‘In some ways, I’m just trying to keep up with other people in my field, do what I think my colleagues are doing, and survive in a culture where a ton of work is expected. . . .’ (Dr Erica Patall, one of the successful early career academics interviewed by Kiewra, Luo, and Flanigan, 2021, p. 2006). Drawing on a latent profile analysis of survey data from 933 PhD students and 190 postdoctoral researchers working at Flemish universities, Boone, Vander Elst, Vandenbroeck and Godderis (2022) found higher risk for burnout among young researchers.

Fleming (2021) offers a bleak look at the consequences of extreme workload in academia and argues that it contributes to the proliferation of conflicts, work-related health issues and widespread discontent in universities. The increased screen time during the pandemic and lack of physical activity further contribute to the risk of burnout among academics (Brandau, Vogt & Garey, 2022). The uncomfortably close relationship between extreme workload and a culture of burnouts point to the need to re-orient the discussion of acceptable workload in academia, especially in light of the intersectionality discussion.

The power relations and chain of influences play out in the choices that are available to academics from marginalised backgrounds. Namely, underrepresented groups are exposed to social discrimination, which leads to experience of more intense pressure to perform and this prolonged stress leads to extra efforts for overperforming, which negatively affects their physical and mental health. Rolle et al. (2021) showed how accumulated stress in the workplace relates to social anxiety and impostor syndrome and results in decreased wellbeing and burnout in STEM academics from racial and ethnic minority groups.

#### Nuanced Narratives of Failure

Kiewra et al.’s (2021) illuminated the successful academics’ ability to respond to failures in constructive terms as they framed failure as a space for growth. I supplement this framing with the need for institutional support when failures occur, especially for marginalised groups. The importance of institutional support is felt most acutely when one has to cope with failures and losses. A typical academic setting is based on hierarchical career progression but has little support for the physical and mental strains endured in the process of climbing the ladder. As one of our interviewees put it: ‘[academia] is like a competitive sport without any support team when you are injured or when it is tough season’. The hierarchical arrangement in academia corresponds to the competitive nature of increasingly neoliberal policies at contemporary universities (Knipfer, Shaughnessy, Hentschel & Schmid, 2017), which manifest in pressure on research staff to secure external funding and higher number of students and classes for teaching staff and increased administrative duties beyond project- or lab-related needs. The policies leave many academics disenchanted and make them re-evaluate their motivations for keeping or applying for academic positions (Gewin, 2022).

It is worth noting here that personal perception of failures vary, and are not necessarily about lost grants or rejected papers. One of our interviewees offered a striking account of the misalignment between external and internal perception of working with influential people at influential places. Although she works as tenured professor at one of the globally top five institutions and has an internationally recognised portfolio of work with lifetime achievement awards, she said that her academic journey was far from challenge-free: ‘I'm not sure looking back on my career that academia was the best place for me. You know, I, I think there is an element of suffering as well’. The quote is a powerful reminder that narratives of success are far more complex than productivity and external assessments. Even within individuals, several identities intersect and navigating their tensions is part of navigating the hidden curriculum of academia.

In conclusion, the recognition that we all employ different strategies to temper our intersecting personal and professional identities can help us address the multiple facets required of individuals to succeed at universities and recognise alternative criteria of academic success. Kiewra et al.’s (2021) quote of Dr. Neugebauer’s passion for education equity suggested the need to define success as pursuing research activities that are in lockstep with one’s values and identities. Hsu et al.’s (2021) interview study indicated that senior leaders with extensive experience in hiring new academic staff based their selection of candidates for tenure-track positions in neuroscience on an eclectic mixture of factors, including scientific vision, quality and potential for collaboration. In our attempt to engage critically with what constitutes success in academia, we asked our interviewees what they consider to be their greatest achievements. Interestingly, neither of the female academics we had interviewed mentioned high productivity rates or publications or any other performance indicators that could be gleaned from their award-studded CVs. Our interviewees framed success in terms of the rewarding aspects of collegiality in the workplace, working at institutions that ‘pursue knowledge and public good’ and the opportunity to ‘watch people grow in their academic career’ through mentoring PhD students and early career researchers (Kucirkova & Fahad, 2022).

The guidance for how to navigate academia should be a shared responsibility directed towards all academics, but especially to those who struggle to visualise their career paths due to systematic inequalities. A serious engagement with intersectional equity brings to fore the disparities that continue to create an uneven playing field for academic women from marginalised groups. Educational psychology, with its explicit focus on education, could model approaches that call out the lack of diverse voices in individual subject areas (e.g. science education, see Zhang, Kirschner, Cobern and Sweller, 2021, in this journal) and promote conditions that facilitate thriving of *all* academics.

## Footnotes

1 Picture a Scientist is a documentary following Biologist Nancy Hopkins, chemist Raychelle Burks, and geologist Jane Willenbring featuring comments from psychologists and researchers from various disciplines aimed at documenting the challenges of women in science and providing new perspectives for more equitable science.
